# Supplementary material for: Asynchronous suppression of visual cortex during absence seizures in stargazer mice
Source: Nat Commun. 2018 May 16;9:1938. doi: 10.1038/s41467-018-04349-8 (PMC5955878; doi:10.1038/s41467-018-04349-8)
Supplement: Supplementary file 1 — Supplementary Information [file 41467_2018_4349_MOESM1_ESM.pdf]

Asynchronous suppression of visual cortex during absence seizures in *stargazer* mice

Meyer et al.

Supplementary information: 1 table and 9 figures

List of recordings and animals used in the study								
**Layer**	**Mouse**	**GCaMP6 type**	**Avg sz length (sec)**	**Avg ISI (sec)**	**# Sz**	**Total time (sec)**	**Total interictal (sec)**	**Total ictal (sec)**
2/3	1	slow	2.9	33.1	46	1453	1319	133
	2	medium	3.9	21.8	143	2381	1837	545
	3	medium	7.0	32.1	47	1240	910	330
	4	medium	4.5	36.0	32	1162	1017	145
	5	medium	3.8	33.7	18	478	415	63
	6	medium	3.1	58.4	25	1105	1028	76
	7	medium	2.2	11.2	119	1587	1323	255
	8	medium	3.5	11.0	95	1471	1036	332
	9	medium	3.6	10.8	88	1249	940	309
4	6	medium	1.4	24.2	132	3434	3175	185
	7	medium	2.7	31.6	40	1336	1232	104
	9	medium	3.1	11.3	107	1533	1192	331
	9	medium	2.7	13.5	53	1126	704	143
	10	medium	3.8	16.6	75	1595	1227	280
5	7	medium	1.6	30.2	50	1575	1478	77
	7	medium	2.0	7.7	157	1533	1202	313
	9	medium	3.2	8.7	132	1460	1072	388
	9	medium	3.4	8.1	135	1562	1080	459
	10	medium	2.5	10.1	122	1548	1223	303
6	8	medium	2.9	15.2	96	1553	1261	211
	8	medium	3.5	11.0	95	1471	1036	332
	10	medium	3.3	8.8	129	1573	1131	424
	11	medium	2.9	6.3	187	1737	1166	547
		**AVERAGE**	**3.2**	**19.6**	**92.3**	**1528.8**	**1217.6**	**273.3**

Supplementary Table 1. Ictal/interictal characteristics and GCaMP6 indicator subtype used.
ISI = inter-seizure interval

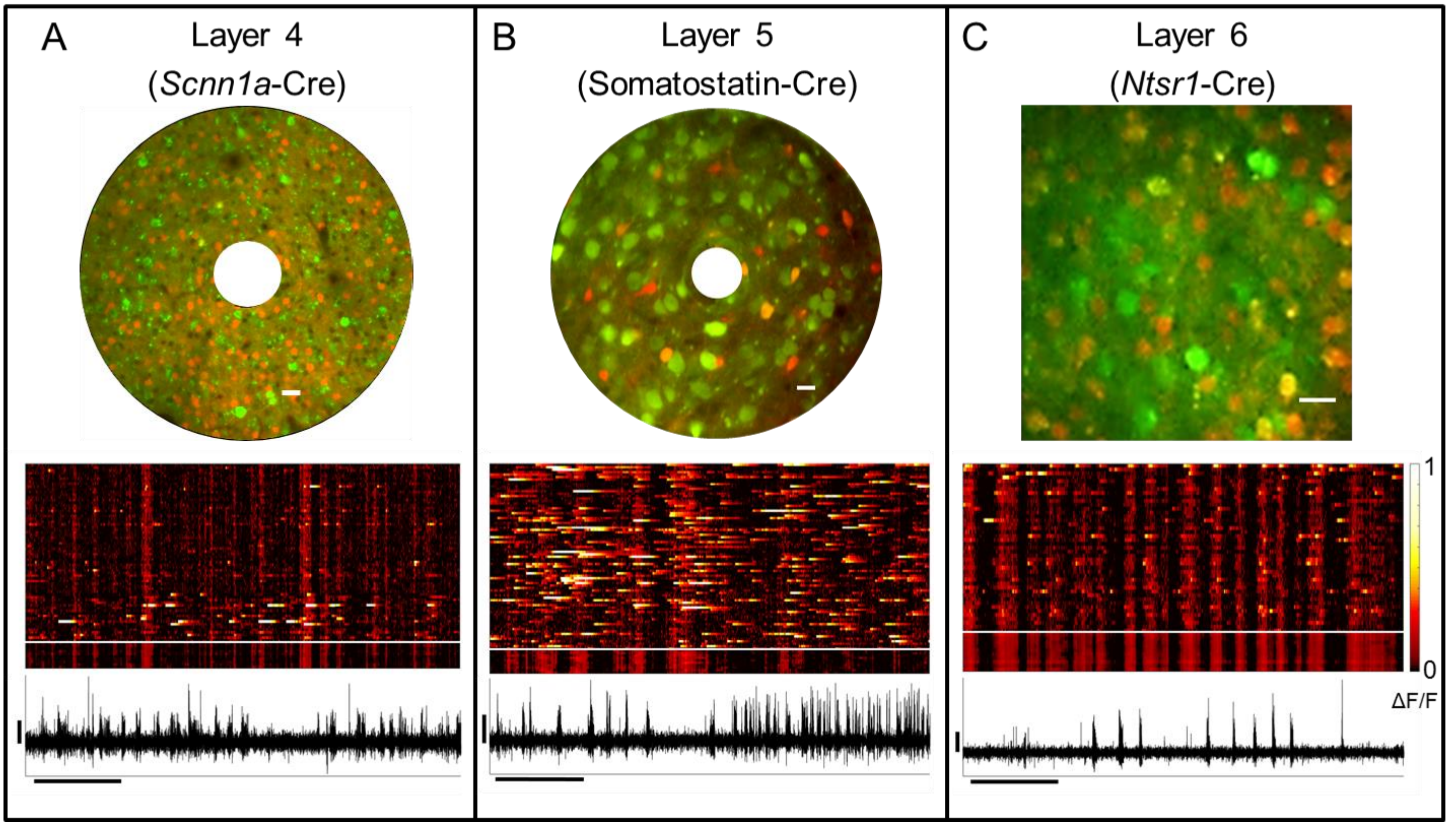

Supplementary Figure 1. Deep layer imaging. Neurons and patches of neuropil were imaged in deeper layers using a combination of depth from the pial surface and layer-dependent expression of tdTomato. A typical field of view for each layer (above), raster plot of activity (middle, neurons above and neuropil below white line) and concomitant EEG (below) are seen in **(A)** Layer 4 (spiral scan), **(B)** Layer 5 (spiral scan), and **(C)** Layer 6 (line scan) of visual cortex (white scale bar = 20 µm, horizontal black scale bar = 10 seconds, vertical black scale bar = 200 µV).

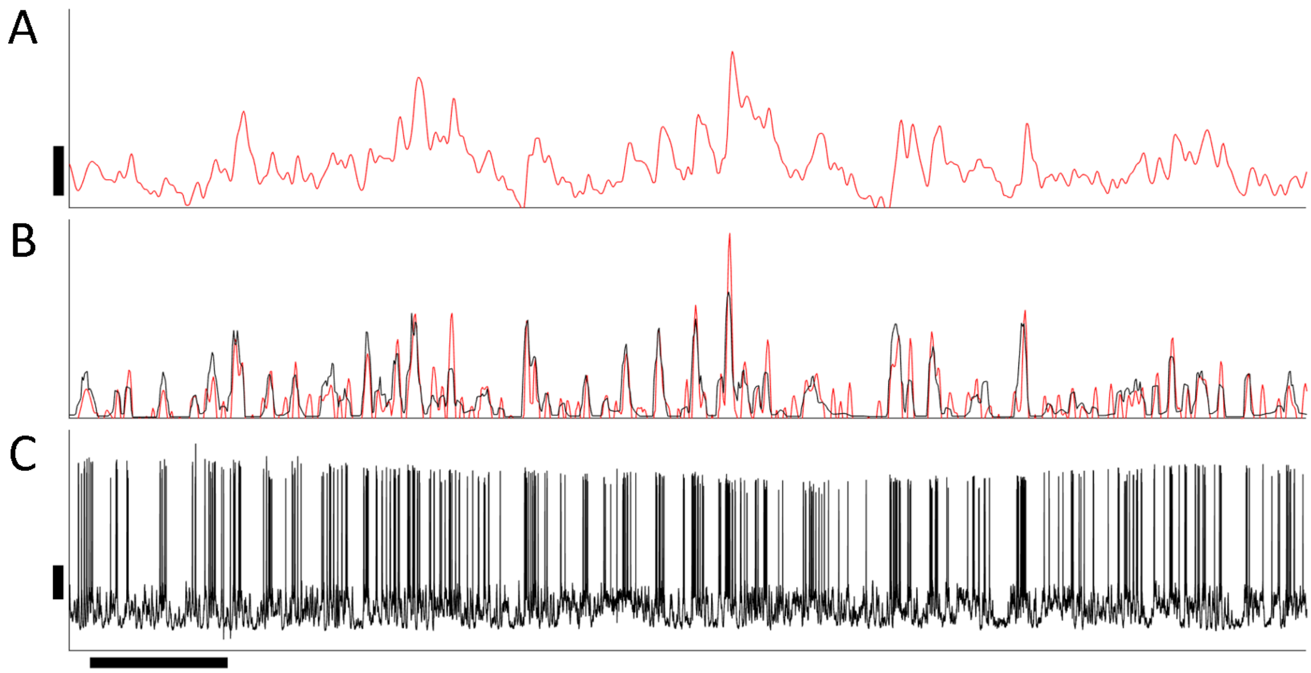

Supplementary Figure 2. Correlation between action potentials and calcium activity in a *stargazer* mouse. A patched, GCaMP6m-filled neuron showing: **(A)** a representative *ΔF/F* trace (vertical scale bar = 20% *ΔF/F*), **(B)** the corresponding deconvolved activity trace (red), the extrapolated firing rate (black, A.U.), and **(C)** the corresponding membrane voltage trace (vertical scale bar: 20 mV). Horizontal scale bar = 10 sec. Correlation coefficients between deconvolved activity and extrapolated firing rates were ~0.8 on average, and the percent of identified action potentials with deconvolved GCaMP6 activity were, on average, 80%, 97%, and 100% for singlets, doublets, and ≥triplets, respectively.

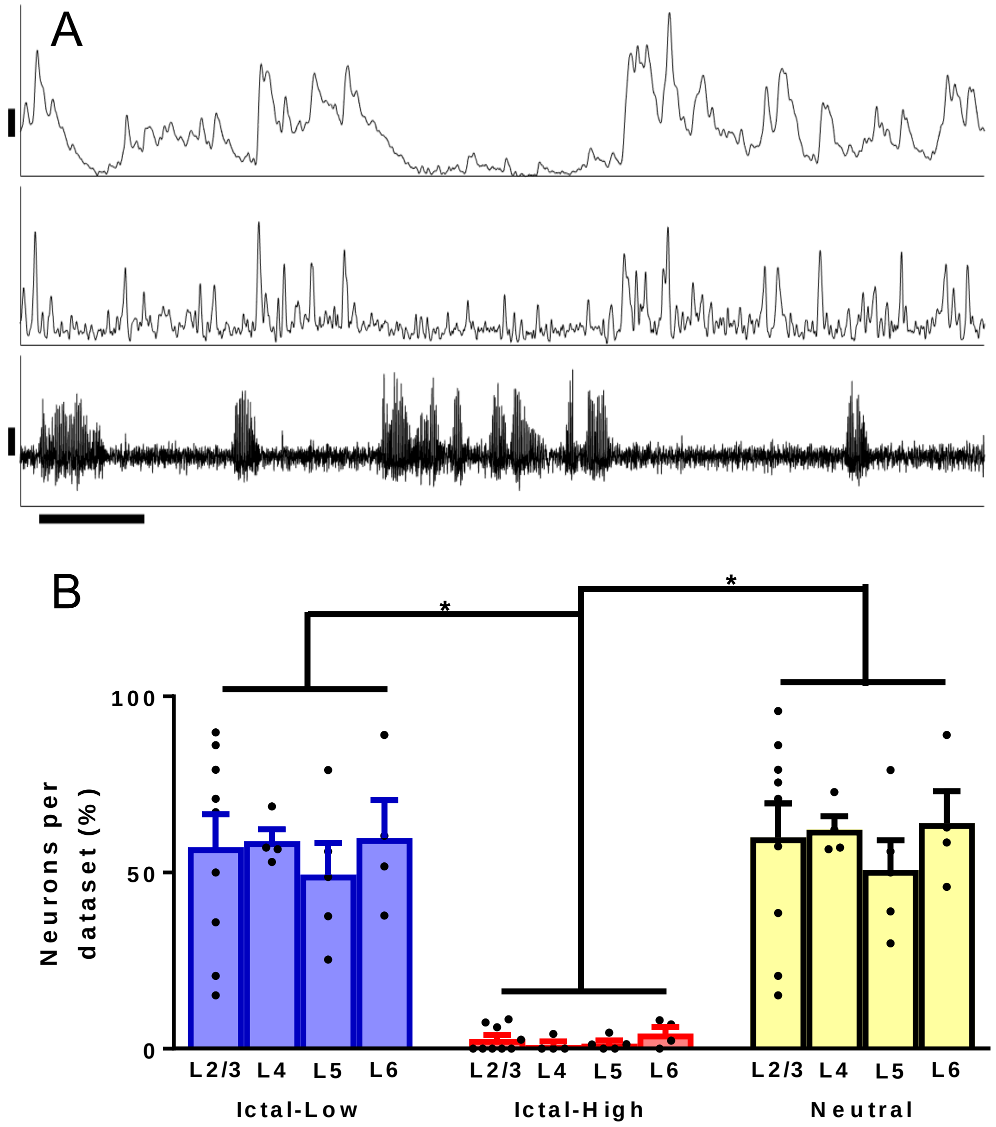

Supplementary Figure 3. Deconvolution of the calcium signal yields similar results. (A) Example of activity traces for a single neuron before and after deconvolution (*top*, *ΔF/F* before deconvolution, vertical bar = 10%); *middle: ΔF/F*, deconvolved, A.U.; *bottom*: simultaneous EEG highlighting that lower activity during seizures can be seen both by analyzing the raw and deconvolved calcium recordings. Vertical bar: 200 µV, horizontal bar = 10 sec. **(B)** Significantly more ictal-low than ictal-high and neutral neurons (bar plots: mean ± s.e.m., one-way ANOVA with post-hoc Bonferroni correction, *p<0.001) are observed after deconvolution. The lower percentage of ictal-low (56.1%) and ictal-high (2.3%) neurons is secondary to the fewer non-zero data points created as a byproduct of deconvolution. Note that in spite of this, the basic conclusions remain unchanged when using deconvolved data.

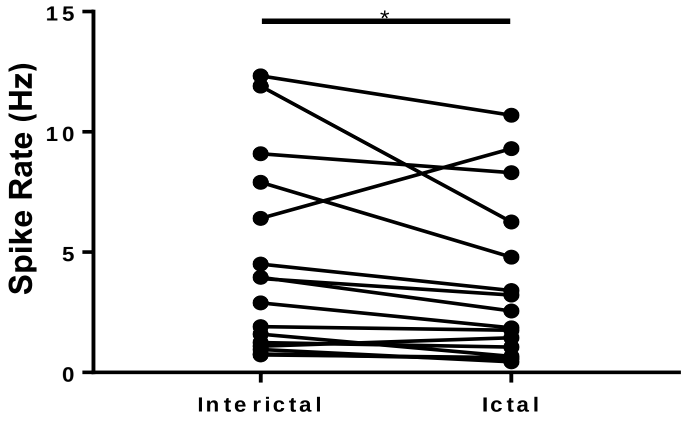

Supplementary Figure 4. Patch-clamp recordings in a subset of animals corroborate the predominantly ictal-low character of L2/3 neurons. Mean ictal and interictal firing rates were calculated from single unit action potentials. 14 of 16 cells were deemed ictal low and 2 cells were ictal high; overall, the neurons were significantly suppressed (**p*=0.0092, Wilcoxon matched pairs signed-rank test).

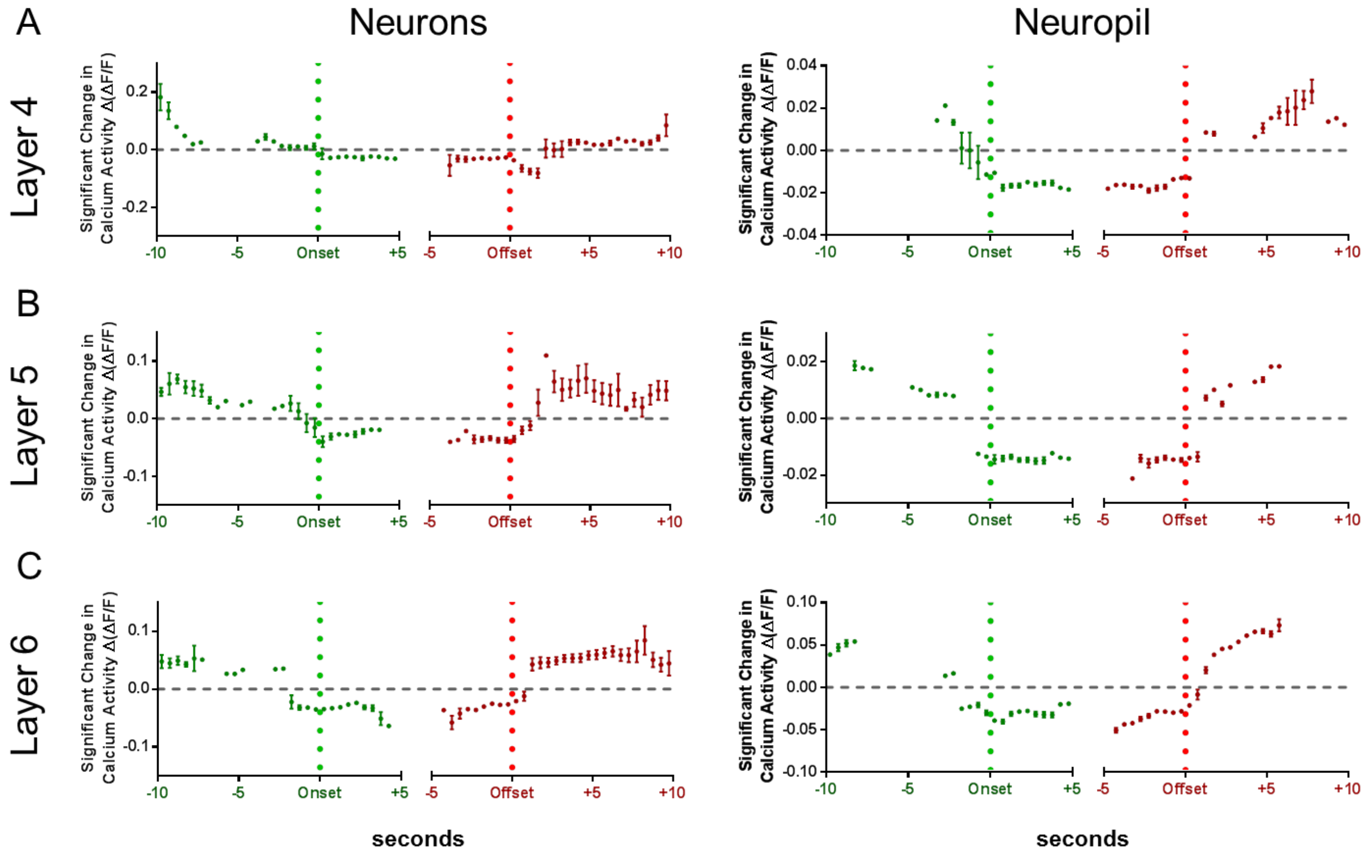

Supplementary Figure 5. Temporal analysis of deep layers is similar to superficial layers. Mean (± s.e.m.) activity change for each 0.5-sec bin aligned to seizure onset (green), and seizure offset (red). In **(A)** Layer 4**, (B)** Layer 5, and **(C)** Layer 6, ictal-low neurons and neuropil consistently reduce activity within a few seconds before onset and after offset. Layer 6 neurons cross below mean activity 2 seconds prior to onset, compared to Layer 4 neurons which cross below mean activity right at seizure onset.

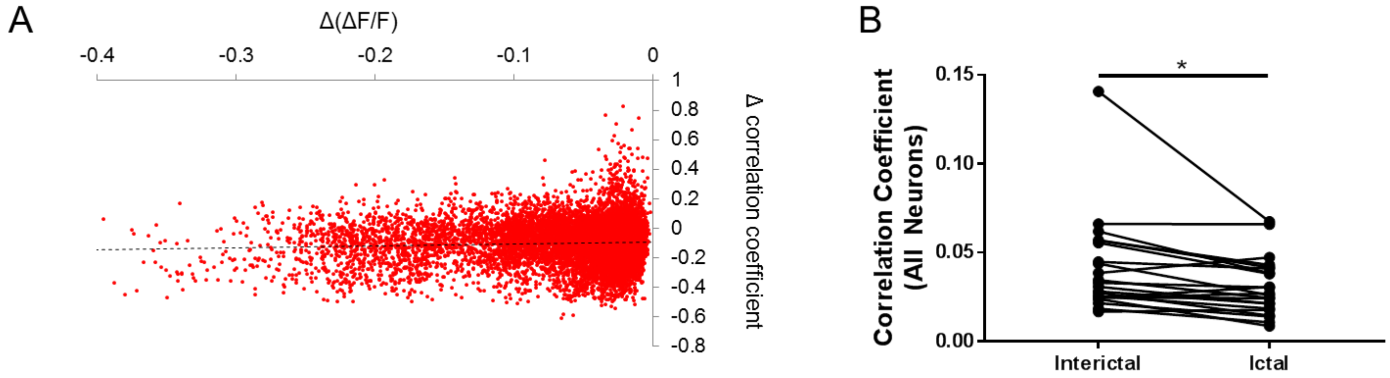

Supplementary Figure 6. Validation of reduced synchrony in the ictal state. (A) There is no significant relationship between the geometric pairwise mean of reduced calcium activity and change in pairwise correlation coefficient (dotted line, r^2=0.003), confirming that the observed decorrelation is not simply due to a change in firing rate. **(B)** Furthermore, after randomly removing activity until deconvolved traces show matching interictal and ictal rates for each neuron, the overall reduction in pairwise correlation in the ictal state remains (**p*=0.0005, Wilcoxon matched pairs signed-rank test).

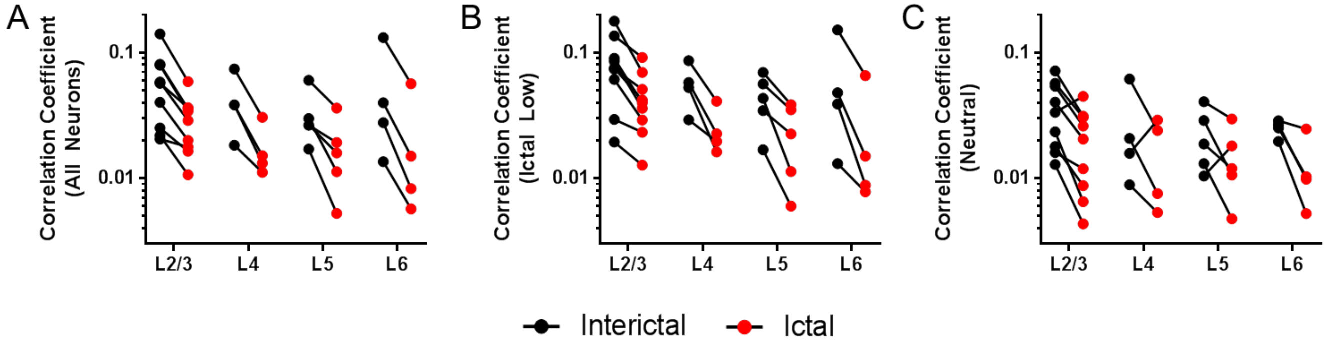

Supplementary Figure 7. Deconvolution of the calcium signal yields similar correlations. Pairwise Pearson correlation coefficients using deconvolved data showed similar reduced correlations during the ictal state, with reductions in overall synchrony **(A)**, most pronounced in ictal-low neurons **(B)**, and a mixture of increased and decreased correlations with neutral neurons **(C)**.

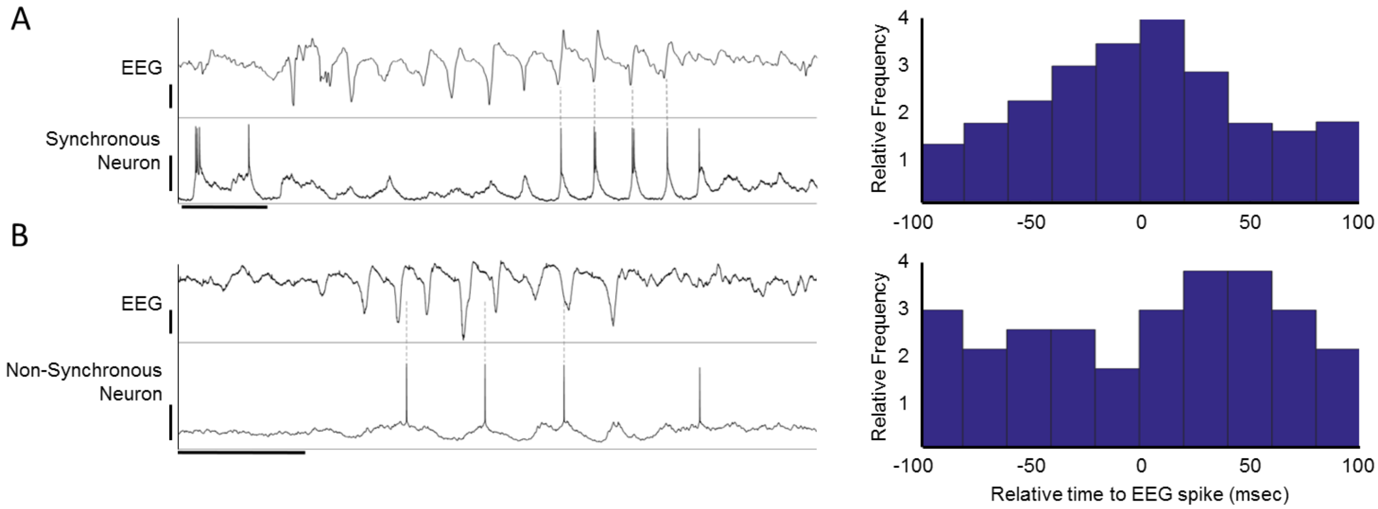

Supplementary Figure 8. Patch-EEG synchrony. Layer 2/3 patched neurons had variable synchrony with EEG spikes. **(A)** Example of relatively high synchrony, with 29.3% of all EEG spikes coinciding with an action potential within ±20 msec. Note that several EEG spikes are not associated with an action potential. **(B)** Example of relatively low synchrony, with only 2.2% of all EEG spikes coinciding with an action potential within ±20 msec. Peri-spike time historgrams for each neuron are shown to the right. Time bar = 0.5 seconds; EEG amplitude bar = 20 µV; Patch amplitude bar = 40 mV. Overall, the likelihood of a neuron spiking within ±20 msec around an EEG spike was 17.9±3.9% (mean±SEM), indicating that synchrony of neurons during spike-wave seizures, measured at high temporal resolution, is low.

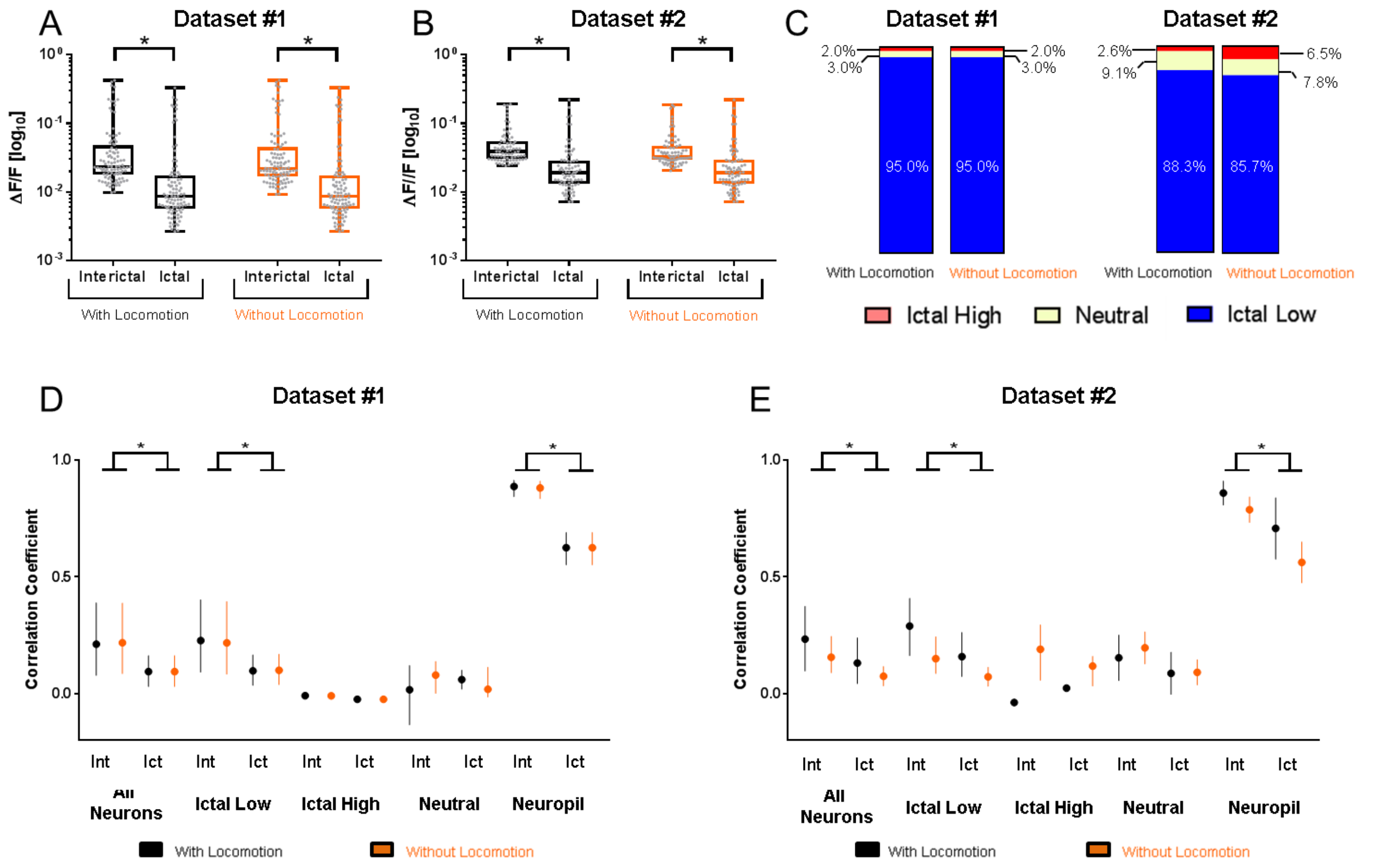

Supplementary Figure 9. No significant change in neuron activity or synchrony when episodes of locomotion are removed (n = 2 datasets). To exclude possible movement contamination, when all imaging frames which occurred during wheel motion are removed, there is no significant change in: reduction in overall activity in neurons (n=100 and n=77 in Dataset #1 **(A)** and #2 **(B)**, respectively; *p<0.0001, Kruskal-Wallis test with Dunn's test for multiple comparisons. Box plots: horizontal line = median, box = 25th – 75th percentile, whiskers = data range), **(C)** relative proportion of ictal low neurons compared to ictal high and neutral neurons, and significant reduction in synchrony of all neurons, ictal low neurons and neuropil in the ictal state in Dataset #1 **(D)** and #2 **(E)**, respectively; *p<0.0001, one-way ANOVA with Bonferroni test for multiple comparisons. Plotted are mean values (± s.e.m.).
